# A Narrative Review on Sarcopenia in Type 2 Diabetes Mellitus: Prevalence and Associated Factors

**DOI:** 10.3390/nu13010183

**Published:** 2021-01-09

**Authors:** Anna Izzo, Elena Massimino, Gabriele Riccardi, Giuseppe Della Pepa

**Affiliations:** Department of Clinical Medicine and Surgery, Federico II University, Via Sergio Pansini 5, 80131 Naples, Italy; ariannaizzo.1991@gmail.com (A.I.); elenamassimino@libero.it (E.M.); riccardi@unina.it (G.R.)

**Keywords:** sarcopenia, type 2 diabetes mellitus, skeletal muscle mass

## Abstract

Type 2 diabetes mellitus (T2DM) represents a major health burden for the elderly population, affecting approximately 25% of people over the age of 65 years. This percentage is expected to increase dramatically in the next decades in relation to the increased longevity of the population observed in recent years. Beyond microvascular and macrovascular complications, sarcopenia has been described as a new diabetes complication in the elderly population. Increasing attention has been paid by researchers and clinicians to this age-related condition—characterized by loss of skeletal muscle mass together with the loss of muscle power and function—in individuals with T2DM; this is due to the heavy impact that sarcopenia may have on physical and psychosocial health of diabetic patients, thus affecting their quality of life. The aim of this narrative review is to provide an update on: (1) the risk of sarcopenia in individuals with T2DM, and (2) its association with relevant features of patients with T2DM such as age, gender, body mass index, disease duration, glycemic control, presence of microvascular or macrovascular complications, nutritional status, and glucose-lowering drugs. From a clinical point of view, it is necessary to improve the ability of physicians and dietitians to recognize early sarcopenia and its risk factors in patients with T2DM in order to make appropriate therapeutic approaches able to prevent and treat this condition.

## 1. Introduction

Type 2 diabetes mellitus (T2DM) is one of the most widespread metabolic diseases. The alarming rise in T2DM prevalence worldwide, exploding in both low-income countries and adolescents/young adults, as well as its heavy impact on longevity and quality of life, poses enormous challenges in the diagnosis, prevention, and treatment of this disease [1]. The improvement of the healthcare system and the advancements in prevention and treatment of major non-communicable illnesses have substantially increased the longevity of the population. Since advancing age represents a risk factor for T2DM and the improvement of health care has contributed to reduced mortality also in the diabetic population, the number of elderly people with T2DM has increased dramatically in recent years [2,3]. T2DM represents an important health burden in the elderly population, affecting approximately 25% of people over the age of 65 years; this proportion is expected to further increase in the next decades [4]. Beyond microvascular and macrovascular complications, elderly people with T2DM have higher rates of functional disability, coexisting illnesses, and several common geriatric disorders [5]. Among these, sarcopenia—a degenerative skeletal muscle condition related to the aging process—has been described as a new complication observed in the elderly population with T2DM [6].

Sarcopenia was defined for the first time in 1988 by Irwin Rosenberg as an age-related decrease in skeletal muscle mass and function [7], and ten years later Baumgartner provided a method for the evaluation of sarcopenia by using appendicular lean mass adjusted for height (kg/m^2^) [8].

From a physiological point of view, a progressive and generalized loss of muscle mass can be observed after the age of 40 years; the rate of deterioration has been estimated to be 8% every ten years up to 70 years, and 15–25% every ten years after this age [9]. Similarly, a 10–15% loss of leg strength every ten years has been reported to occur up to 70 years, increasing to 25–40% every ten years after this age [10]. Despite the clear notion of sarcopenia, its current evaluation in clinical practice is still not univocal. This gap is strictly related to the fact that evaluation of muscle strength, mass, quality, and performance should comprehensively be taken into account for the definition of sarcopenia.

In recent years, different definitions by international consensus have been proposed. In 2010, the European Working Group on Sarcopenia in Older People developed a practical clinical definition and consensus diagnostic criteria for sarcopenia recommending to utilize the presence of both low muscle mass and low muscle function (strength or performance) [11]. In 2018, an update by the same working group was provided defining sarcopenia by low measurement levels of three parameters: muscle strength (evaluated by hand grip strength), muscle quantity/quality (evaluated by appendicular lean mass or skeletal muscle mass), and physical performance (evaluated by gait speed or short physical performance battery) as an indicator of severity [12]. Similarly, the Asian Working Group for Sarcopenia developed its own consensus—the only difference being in cut-off values, in consideration of the anthropometric characteristics of the Asian population [13]. Reduction in muscle mass and physical performance were included in the definition of sarcopenia by the International Working Group on Sarcopenia [14], while reduction in muscle mass and strength were included in the diagnostic criteria proposed by the American Foundation for the National Institutes of Health [15].

The prevalence of sarcopenia worldwide ranges from 10% to 40%, based on the different characteristics of the population and the different criteria to asses it [16]. In non-Asian countries, the prevalence is higher than in Asian ones, without differences between genders [17]. Furthermore, the prevalence of sarcopenia is higher in acutely hospitalized older patients [18] or in patients admitted to post-acute geriatric rehabilitation [19]. In fact, sarcopenia is associated with poor health outcomes—such as cognitive impairment and functional decline [20,21], depression [22], falls and fractures [23], and mortality [21]—and can be considered the main physical drivers of frailty or perhaps even a precursor state. Sarcopenia and frailty are strictly related and lead to difficulties performing daily living activities and increased disability [24]. Frailty is a condition in which the individual is highly vulnerable to stressors that lead to disability, dependency, and mortality with decreased physical reserves [25,26]. The most common definition used for frailty is the frailty phenotype, consisting of five physical components to define frailty: unintentional weight loss, self-reported exhaustion, weakness, slow walking speed, and low physical activity [27]. Individuals are considered to be frail when they meet three or more criteria, and they are considered to be robust when they have none. The Frailty Index is another popular approach describing frailty as a state caused by the accumulation of health deficits during the life course. It is calculated as the ratio of the number of deficits present in the individual to the number of total deficits considered [28]. The Edmonton Frail Scale (EFS) [29], its recent validated short version (EFS-SF) [30,31], the Fatigue, Resistance, Ambulation, Illnesses, and Loss of Weight (FRAIL) Score [32], the Geriatricians’ Clinical Impression of Frailty (GCIF) scale [33], and the Clinical-Functional Vulnerability Index-20 (IVCF-20) [34] are other approaches able to identify frailty.

It is important to underline that the progressive increase in obesity worldwide configures a new public health problem: sarcopenic obesity, a condition in which sarcopenia and obesity are coexisting [35]. Sarcopenic obesity might be related to different factors such as aging, reduction in physical activity, malnutrition, sub-clinical inflammation, and hormonal changes [36]. Dramatically, the clinical consequences of sarcopenic obesity are much greater than sarcopenia alone; in fact, several studies reported that sarcopenic obesity is associated with higher fasting blood glucose, insulin resistance, blood pressure, plasma lipid abnormalities, T2DM, cardiovascular diseases, and mortality compared with sarcopenia or obesity alone [35,37,38,39].

Sarcopenia has received growing interest over the last decade—it is now recognized as an independent condition by the International Classification of Disease, Tenth Revision, Clinical Modification (ICD-10-CM), code (i.e., M 62.84) [40]—particularly for the progressive increase in life expectancy, for its global diffusion, and for the strong association with several chronic diseases [41]. It is estimated that during the next 30 years, the prevalence of sarcopenia will significantly rise [42], making it a major public health issue.

Currently, increasing attention has been paid to sarcopenia in T2DM individuals, for both clinical and research reasons, due to the heavy impact that sarcopenia may have on the quality of life of elderly people with T2DM [43], in which frailty and sarcopenia are emerging as a third category of complications, in addition to the traditional microvascular and macrovascular disease leading to considerable disability [44].

Particular interest is focused around two issues: (1) whether sarcopenia is associated with features of the diabetic condition such as metabolic control, disease duration, and presence of diabetes complications, and (2) the role played by diet and glucose-lowering drugs in the development of sarcopenia. Information on these issues is relevant in order to better identify patients at higher risk for sarcopenia and to implement appropriate dietetic and pharmacological strategies to reduce this risk [45].

Against this background, the aim of the present narrative review is to provide an update on: (1) the risk of sarcopenia in individuals with T2DM and (2) its association with relevant features of T2DM patients such as age, gender, body mass index (BMI), disease duration, glycemic control, presence of microvascular or macrovascular complications, nutritional status, and glucose-lowering drugs.

## 2. Materials and Methods

We conducted a narrative review [46] by searching PubMed databases for articles published during the last 20 years (2000–2020), also considering, if available, previous most relevant studies, about the prevalence of sarcopenia in T2DM and its associated factors. The search dated from April 2020 to November 2020.

The medical subject heading (MeSH) terms “sarcopenia” AND “diabetes” OR “type 2 diabetes mellitus” OR “diabetes mellitus” OR “blood glucose control” OR “glycated hemoglobin” OR “diabetes treatment” OR “diabetes diet” OR “glucose-lowering drugs” OR “diabetes complications” OR “microvascular complications” OR “macrovascular complications” were utilized.

The search was limited to humans and the English language. With respect to article types, prospective cohort studies, retrospective and cross-sectional studies, meta-analyses, and systematic reviews on epidemiological studies were included in this narrative review. Furthermore, existing editorials and commentaries to the most relevant articles were also considered.

Conversely, studies that did not meet the selection criteria, duplicate publications, non-original articles, and studies in languages other than English were excluded. For the aim of our narrative review on prevalence/associated factors, we also excluded interventional clinical trials.

Titles and abstracts of retrieved studies were screened to select potentially relevant articles. Full texts were analyzed independently to determine whether they met the established inclusion criteria. Initially, 749 articles of potential intertest were found. After screening of titles and abstracts, 56 papers were used for the aim of this narrative review.

## 3. Results

### 3.1. Prevalence of Sarcopenia in Individuals with Type 2 Diabetes Mellitus

In epidemiological studies, the prevalence of sarcopenia in T2DM has a wide range, varying from 7% to 29.3% in different populations [6,47,48,49,50,51,52,53,54,55,56,57,58,59,60,61,62,63,64,65,66,67], (Table 1).

In T2DM, a higher prevalence of sarcopenia has been consistently reported in respect to normoglycemic individuals. In particular, a very recent meta-analysis, including a total number of 6526 participants (1832 with T2DM and 4694 controls, and 1159 cases of sarcopenia), showed that the prevalence of sarcopenia is significantly higher in T2DM than in non-diabetic individuals (OR 1.55; 95% CI 1.25–1.91; *p* < 0.001) [68]. The prevalence of sarcopenia ranged from 5% to 50% in the 15 studies analyzed (seven studies were cross-sectional and eight were case-control, respectively). Interestingly, T2DM individuals presented lower muscle performance and strength compared with the normoglycemic ones, whereas no difference in muscle mass was observed [68]. Similarly, Veronese et al. in their meta-analysis reported that the prevalence of sarcopenia in T2DM was 28.4% vs. 18.7% in the control group (OR 1.63; 95% CI 1.20–2.22; *p* = 0.002) [69].

### 3.2. Sarcopenia in Individuals with Type 2 Diabetes Mellitus: Associated Factors

#### 3.2.1. Age

**Figure 1:**
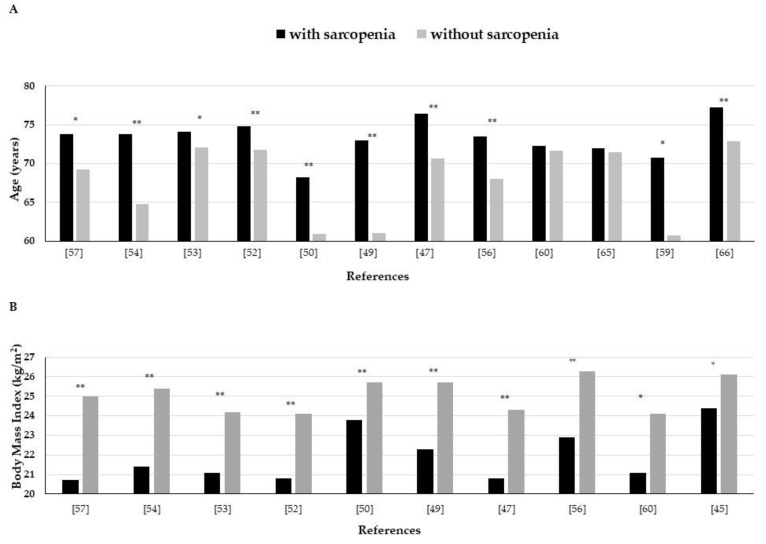
Mean values of age (**A**) and body mass index (**B**) in type 2 diabetes mellitus individuals with and without sarcopenia in epidemiological studies. * *p* < 0.05; ** *p* < 0.001.

A significant association between sarcopenia and age in T2DM individuals has been repeatedly reported. Most of the studies have shown that individuals with T2DM and sarcopenia on the average are older than those without sarcopenia: 73.6 years and 67.2 years, respectively (Figure 1A). In fact, as observed by Cui et al., the prevalence of sarcopenia in T2DM rises progressively by increasing age (17.4% in the age group 65–69 years, 28.1% in the age groups 70–74 years, 52.4% in the age groups 75–80 years, and 60% in the age groups above 80 years) [56]. Similarly, Murata et al. showed that approximately 40% of T2DM patients ≥80 years old present with sarcopenia [67].

#### 3.2.2. Gender

The impact of gender on the prevalence of sarcopenia differs among studies and data are not univocal. In some studies, a significantly higher prevalence of sarcopenia in men [48,63,64] or women [65,70] was reported. However, other studies [47,49,50,51,52,53,54,56,57,58,59,60,61,62,66] did not find any difference in gender distribution among T2DM with sarcopenia.

Furthermore, Anagnostis et al. reported that the point estimate for sarcopenia risk in men and women with T2DM was 1.72 (95% CI 1.1–2.69; *p* = 0.017) and 1.46 (95% CI 0.94–2.25; *p* = 0.08), respectively, compared with those without T2DM, and this difference between genders was not significant [68].

#### 3.2.3. Body Mass Index

BMI is significantly lower in T2DM individuals with sarcopenia compared with those without it and many studies have shown that the prevalence of sarcopenia significantly decreases as BMI increases (Figure 1B). Interestingly, Fukuoka et al. showed that grouping T2DM individuals in quartiles of body fat, the lowest prevalence of sarcopenia was observed in the third quartile as compared with both the first and the fourth quartiles [66]. This finding suggests that in T2DM individuals, both a low BMI and high body fat are associated with sarcopenia.

#### 3.2.4. Diabetes Duration

**Figure 2:**
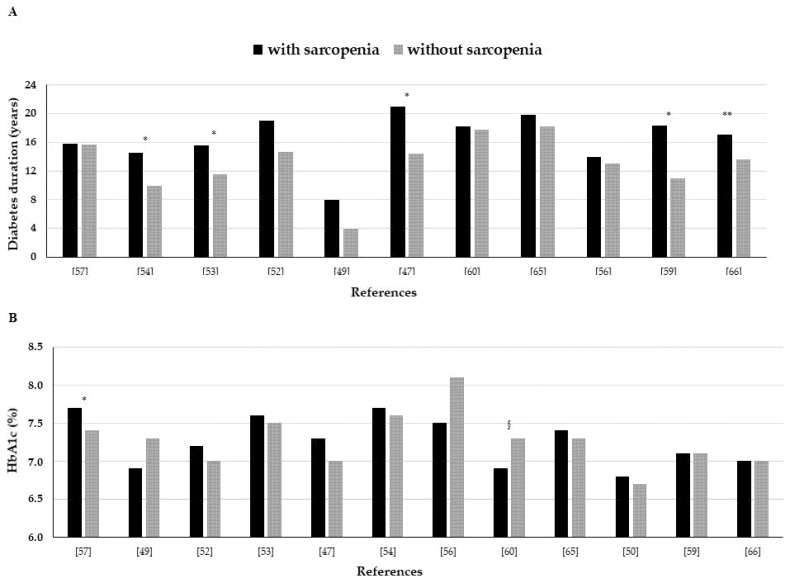
Mean values of diabetes duration (**A**) and glycated hemoglobin (**B**) in type 2 diabetes mellitus individuals with and without sarcopenia in epidemiological studies. * *p* < 0.05; ** *p* < 0.001; ^§^ *p* < 0.05 in men.

Looking at T2DM duration, data are not univocal. In some studies, the longer the duration of diabetes, the higher the prevalence of sarcopenia [47,53,54,59,66]. In particular, the paper by Cui et al. showed that dividing participants according to diabetes duration, the prevalence of sarcopenia was 27.6%, 21.8%, and 52.6%, respectively in the groups with a diabetes duration below 10, between 10 and 20, and above 20 years [65]. However, other studies [49,52,56,57,60,65] did not find any association between disease duration and sarcopenia (Figure 2A). This was confirmed by the meta-analysis of Anatagostis et al. showing no difference in sarcopenia prevalence between individuals with a mean T2DM duration of ≥9 years or <8.5 years [68].

#### 3.2.5. Glycemic Control

In epidemiological studies, glucose control, evaluated mainly by measuring glycated hemoglobin (HbA1c), has not been found associated with sarcopenia in TD2M individuals (Figure 2B). Only in few studies the frequency of sarcopenia was related to HbA1c levels [57,71]. Among them, Sugimoto et al. reported that the frequency of sarcopenia increases linearly with HbA1c, particularly in lean individuals, and this association is independent of major covariates, including anthropometric factors and duration of diabetes [57]. Contrarily, Ida et al. reported lower HbA1c levels in men with sarcopenia [60].

#### 3.2.6. Microvascular and Macrovascular Complications

Retinopathy, nephropathy, and neuropathy are common microvascular complications of T2DM. In some studies, a difference in the prevalence of sarcopenia according to the presence of these complications has been observed. Fukuda et al. reported that sarcopenia and low muscle strength were significantly associated with the progression of diabetic retinopathy, and in particular, proliferative retinopathy (OR 7.78, 95% CI 1.52–39.81, *p* = 0.014, and OR 6.25, 95% CI 1.15–33.96, *p* = 0.034, respectively) [63].

Although the frequency of nephropathy was similar in T2DM individuals with and without sarcopenia [49,57,65,71], Bouchi et al. showed that in the presence of sarcopenia the risk for progression of albuminuria was increased [49].

With respect to neuropathy, Ida et al. reported that it was more frequent among women than among men in the sarcopenic group [51]. Nomura et al. reported that in men and women aged >50 years, individuals with neuropathy had lower knee extension strength relative to body weight compared with individuals without neuropathy [72]; similar findings were reported by Kalyani et al. [73].

**Figure 3:**
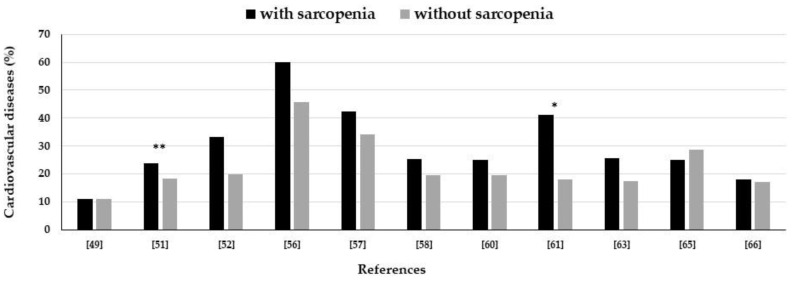
Prevalence of cardiovascular diseases in type 2 diabetes mellitus individuals with and without sarcopenia in epidemiological studies. * *p* < 0.05 in people with high visceral fat; ** *p* < 0.001 in women.

Epidemiological studies have not shown differences in the prevalence of cardiovascular diseases in T2DM patients with or without sarcopenia (Figure 3). In one study, Ida et al. observed a higher prevalence of cardiovascular disease in T2DM women with sarcopenia [51]. Interestingly, the paper by Murai et al. showed that in T2DM individuals with sarcopenia, lower muscle quality and high visceral fat were significantly associated with cardiovascular diseases as compared to the presence of lower muscle quality and low visceral fat; this suggests that T2DM individuals with visceral fat accumulation and low muscle quality have a higher cardiovascular risk [61].

With respect to diabetic foot disease, a common long-term complication of diabetes, Cheng et al. reported that the percentage of sarcopenia in diabetic foot disease patients was more than double than those without diabetic foot disease, and sarcopenia was indipendently associated with this condition [74]. Furthermore, in the diabetic foot disease group, patients with sarcopenia exhibited more foot ulcers, higher Wagner grade, and greater percentage of amputation compared with patients without sarcopenia [74].

#### 3.2.7. Nutritional Status and Lifestyle

A high prevalence of sarcopenia in T2DM individuals with a poor nutritional status has been observed [75].

In particular, Okamura et al. reported that in T2DM patients with sarcopenia the energy intake was significantly lower than in those without sarcopenia (1499 vs. 1786 kcal/day, respectively), whereas no difference in the intake of proteins, carbohydrates, or fats was observed. After adjusting for age, sex, exercise, smoking status, HbA1c, and BMI, patient’s energy intake was negatively associated with the presence of sarcopenia (OR 0.86; 95% CI 0.78–0.95; *p* = 0.001) [53].

In T2DM individuals with sarcopenia, the omega-3 fatty acids intake has been found to be reduced in comparison with those without sarcopenia (2.6 vs. 3.0 g/day, respectively) [76].

Regarding other lifestyle factors, epidemiological studies do not report differences in smoking and drinking habits between T2DM individuals with and without sarcopenia (Table 2), also when the analysis was performed separately in men and women [51,60]. Conversely, in T2DM individuals with sarcopenia the prevalence of physical activity has been found to be lower [56,64,77]; similarly, walking >5401 steps/day has been shown to have a protective effect since it is associated with a 70% lower prevalence of sarcopenia in T2DM patients [64].

#### 3.2.8. Glucose-Lowering Drugs

In most studies evaluating the association between different glucose-lowering drugs and the prevalence of sarcopenia the majority of the participants were on at least two medications; the most frequently used were biguanides (metformin), dipeptidyl dipeptidase 4 inhibitors, sulfonylureas, and insulin (Table 3). Overall, the use of glucose-lowering drugs did not differ significantly between patients with or without sarcopenia, except for biguanides that were less frequently used in patients with sarcopenia [49,56,66,77]. Considering possible gender differences, the inverse association between biguanide use and sarcopenia was more marked in men [66].

## 4. Discussion

The evidence presented in this narrative review shows that the prevalence of sarcopenia in individuals with T2DM is high and widely ranges from 7% to 29.3%. The wide variation in sarcopenia prevalence could be due to different reasons such as the diagnostic criteria to assess it (muscle strength, muscle quantity/quality, and physical performance), as well as the different methods for its quantitative evaluation (dual-energy X-ray absorptiometry or bio-electrical impedance analysis). Moreover, the prevalence of sarcopenia in T2DM individuals may depend on the characteristics of the population such as ethnicity—most of the studies were carried out in Asia—age, BMI, or associated comorbidities.

The higher prevalence of sarcopenia in T2DM individuals may be explained by different mechanisms. The anabolic action of insulin in skeletal muscle is well known; it may be progressively lost in T2DM due to the impaired insulin sensitivity associated with this disease. In addition, the impairment in insulin action may induce a decreased protein synthesis and an increased protein degradation leading to a reduction in muscle mass and strength [78].

Furthermore, chronic hyperglycemia promotes per se the accumulation of advanced glycosylation end products (AGEs) in skeletal muscle, and a correlation between AGEs and reduction in grip strength, leg extension power, and slow walking speed has been reported [79]. Again, diabetes is associated with an increase in some inflammatory cytokines that may act on muscle loss and favor a reduction in muscle strength and function [45].

Finally, the higher prevalence of sarcopenia in T2DM is also related to the presence of macrovascular or microvascular complications, i.e., retinopathy, nephropathy, and neuropathy. In particular, chronic kidney disease secondary to diabetic nephropathy may affect muscle mass [80], peripheral diabetic neuropathy may impair its strength [72], together to a reduced physical activity and performance due to unstable postural balance or impaired vision [81]. Macrovascular complications, i.e., peripheral vascular disease, may also contribute by inducing muscle ischemia, as well as lower muscle strength, mass, and performance [82].

A significant association between sarcopenia and age in T2DM individuals has been repeatedly reported. It is not surprising that increasing age represents a risk factor for sarcopenia in T2DM, as well as in the general population, due to the age-related decline in skeletal muscle mass and strength as well as in physical performances.

It would be interesting to understand if the factor of age could be more determinant to sarcopenia development in individuals with T2DM compared with those without T2DM. In this regard, Tamura et al. showed no difference on sarcopenia risk according to age categories between individuals with and without T2DM [83]. Similarly, Çeliker et al. reported a higher prevalence of sarcopenia in T2DM individuals (21.4%) compared with those without T2DM (15.1%), while no significant differences in terms of age were observed [71]. Again, Trierweiler et al. reported a higher prevalence of sarcopenia in T2DM individuals (15.6%) compared with those without T2DM (2.4%), with a similar age between groups [84]. Interestingly, the meta-regression analysis (using as moderators mean age and the difference in mean age between individuals with T2DM and controls) performed by Veronese et al.—ten studies were included, showing an increased prevalence of sarcopenia in DT2M compared with controls—did not explain any of the differences found in the sarcopenia prevalence between groups with regard to age [69].

The impact of gender on sarcopenia prevalence in T2DM differs among studies, similarly to the general population, in which some studies showed a higher prevalence among men [85,86], whereas others depicted a higher prevalence among women [87,88]. With regard to sarcopenic obesity, a higher prevalence in men than in women was observed [38,89,90], probably related to the muscle decline in men and a fat increase in women during aging [90], as well as to hormonal changes [36]. Interestingly, women with sarcopenic obesity were more likely to have higher blood glucose, whereas men were more likely to have osteoporosis and dyslipidaemia [90].

BMI is significantly lower in T2DM individuals with sarcopenia compared with those without it. Notably, it is important to underline that individuals with T2DM and sarcopenia have normal weight (Figure 1B), despite the rising increase in obesity in T2DM. This surprising data could be related to the fact that most of the studies were carried out in Asia. Generally, Asians tend to have a relative lower BMI, a higher fat mass, and a lower prevalence of sarcopenic obesity than Caucasians [91,92]. Similarly, Asians with T2DM had lower BMI compared with Caucasians [93]. Furthermore, an increase in abdominal fat can be observed with aging, and this phenomenon might occur even without significant changes in BMI or body weight [94].

Therefore, an evaluation of obesity in elderly T2DM with sarcopenia should not be judged by BMI alone; rather, it should be considered in combination with other adiposity indices—i.e., waist circumference—or evaluation of body fat content [95].

Looking at T2DM duration, data are not univocal. The higher prevalence of sarcopenia observed in T2DM individuals with a longer duration of diabetes reported in some studies could be explained by the effect of chronic hyperglycemia on muscle mass/quality [96] or by the increased prevalence of diabetes complications associated with a longer disease duration [45,97].

In epidemiological studies, glucose control, evaluated mainly by HbA1c, has not been found associated with sarcopenia in T2DM individuals. It is important to underline that although some studies have reported that HbA1c levels are not associated with impaired muscle quality [98], muscle strength [99], and physical performance [73], a relationship between glucose control and sarcopenia cannot be completely excluded; in fact, HbA1c levels do not adequately reflect all the aspects of the day-to-day blood glucose profile and, in particular, the occurrence of hypoglycaemic episodes and the daily glucose fluctuations that may potentially represent risk factors for sarcopenia [100].

In relation to diabetes complications, there are some indications that sarcopenia could be associated with diabetic retinopathy, neuropathy, and foot disease. This last condition might further impair the quality of life of these patients, as well as increase the social and economic burden, cognitive impairment, gait disorder, depression, and morbidity [101,102]. Drammatically, sarcopenia increases the risk of mortality in patients who undergo amputation for a diabetic foot [103].

Looking at the nutritional aspects, it is widely known that malnutrition is one of the risk factors for the development of sarcopenia [12,104]. In the studies reviewed above, the intake of energy and omega-3 fatty acids are significantly reduced in T2DM patients with sarcopenia in comparison with those without this condition. It has been reported that a reduced energy intake, even with an appropriate supply of proteins, may cause a reduction in the protein synthesis in the human body [105]. With regard to omega-3 fatty acids, the explanation of this finding is not straightforward; however, it has been proposed that omega-3 fatty acids can reduce inflammation, thus improving the strength of skeletal muscle [106]. Moreover, they can stimulate muscle protein synthesis [107] and improve the overall neuromuscular function [108].

With regard to other dietary components, further aspects should be explored in T2DM with sarcopenia; in fact, in non-diabetic individuals, there are some promising data regarding the role of vitamin D and sarcopenia, but it is unclear whether the dose, frequency of dose, or length of treatment impacts the efficacy of vitamin D on improving muscle mass or function [109]. Furthermore, selenium and magnesium have been studied as supplements in clinical trials and in the diet, and they appear to demonstrate a potential association with physical activity, muscle performance, and bone health in older individuals [109,110]. Following the Mediterranean diet and higher consumption of fruits and vegetables have been associated with improved physical performance and protection against muscle wasting, sarcopenia, and frailty [109]. The supplementation of the branched-chain amino acid, leucine, or leucine-enriched protein is one of the most common interventions for treating sarcopenia in older individuals [111,112], and evidence reported that administration of leucine or leucine-enriched proteins (range 1.2–6 g leucine/day) is well-tolerated and significantly affects sarcopenia in elderly individuals, improving muscle strength, lean mass content, and walking speed although these effects depend on sarcopenia criteria and the patients’ characteristics [113].

A further interesting aspect to explore should be the intake of coffee—the most consumed psycho-active substance in the world—on sarcopenia in T2DM patients. In fact, evidence has shown that higher consumption of coffee, both caffeinated and decaffeinated, was associated with a significant lower risk of T2DM [114]; in particualr, the risk of T2DM decreased by 6% for each cup-per-day increase in coffee consumption [115]. Epidemiological data from the Korea National Health and Nutrition Examination Survey suggested that consuming at least 3 cups of coffee per day was associated with a lower prevalence of sarcopenia [116], and Kim et al. reported that men who drink one cup of coffee per day were less prone to developing sarcopenia compared to those who rarely drink coffee [117]. The plausible protective role of coffe on sarcopenia might be related to its components with anti-inflammatory and antioxidant properties [118].

Regarding lifestyle factors, epidemiological studies reported that in T2DM individuals with sarcopenia, the prevalence of physical activity is lower. It is important to underline that the kind and the level of physical activity was variable among the studies and defined as: to perform any kind of sport regularly at least once a week [47,52,53,54], to count number of steps/day [56,57], to perform 150 min/week of moderate-intensity aerobic physical activity (50–70% of maximum heart rate) [77].

Similarly, also in individuals without T2DM, the prevalence of physical activity has been found to be lower. In the AGES–Reykjavik study, 53.8% of sarcopenic individuals did not engage in physical activity compared with 37.3% of non sarcopenic individual [119]; Kim et al. reported that only 24% of severely sarcopenic individuals engaged phisical activity compared with 42.8% of non-sarcopenic individuals [120]. The beneficial effect of physical activity on sarcopenia might be related to the improvement in muscle mass, strength, and function [105].

As for the glucose lowering drugs, biguanides might have a protective role for the development of sarcopenia, as indicated by observational cross-sectional studies; however, data from prospective or intervention trials are lacking. The presumed protective role of metformin in relation to sarcopenia may be explained by some possible actions of this drug such as the effect on insulin sensitivity—involved in the uptake of glucose and calcium in skeletal muscles [121]—and the reduction of inflammation and oxidative stress, which may lead to an improvement of muscle quality and strength [122,123].

## 5. Conclusions

The available evidence is rather concordant in showing that the prevalence of sarcopenia in T2DM patients is increased. Different mechanisms may be responsible for this association such as impaired insulin sensitivity, chronic hyperglycemia, AGEs, subclinical inflammation, and microvascular and macrovascular complications. However, only increased age and a low BMI have been demonstrated to represent major risk factors for sarcopenia in T2DM. Information on other features of T2DM associated with this condition are inconsistent; nevertheless, overall, glucose control and diabetes duration do not seem to play a major role. In relation to diabetes complications, there are some indications that sarcopenia could be associated with diabetic retinopathy, neuropathy, and foot disease, while no association with macrovascular complications has been observed.

Among the lifestyle factors, the intake of energy and omega-3 fatty acids as well as the habitual physical activity are significantly reduced in T2DM patients with sarcopenia in comparison with those without this condition. As for the glucose-lowering drugs, biguanides might have a protective role for the development of sarcopenia, as indicated by observational cross-sectional studies; however, data from prospective or intervention trials are lacking.

In conclusion, the increased prevalence of sarcopenia in the elderly population with T2DM and its heavy impact on their quality of life, affecting physical and psychosocial health, make it a major public health issue. Major research gaps in this issue include the relationship of sarcopenia with relevant features of T2DM and its treatment as well as the possibility to prevent it by appropriate lifestyle interventions. From a clinical point of view, it is of paramount importance to increase the ability of physicians and nutritionists to recognize the presence of sarcopenia and its risk factors in T2DM, particularly in older patients with a low BMI; this would allow the implementation of appropriate therapeutical strategies focused on adequate energy intake and regular physical activity. On the other hand, a more effective—although not at all easy—approach to reduce the prevalence and the severity of sarcopenia in the elderly population would be the implementation of lifestyle modifications known to be effective in the prevention of T2DM.

## Figures and Tables

**Table 1:** Prevalence of sarcopenia in patients with type 2 diabetes mellitus.

Reference	Study Design	Participants	BMI (Kg/m^2^)	HbA1c (%)	Sarcopenia Assessment	Sarcopenia Prevalence
[47]	cross-sectional	144 M/W 71.4 years	23.9 ± 3.6	7.1 ± 0.7	AWGS	11.8%
[48]	cross-sectional	506 M/W 67.6 years	27.5 ± 8.1	8.1 ± 2.1	AWGS	28.5%
[49]	retrospective	238 M/W 67 years	24 ± 4.1	7.1 ± 1.3	AWGS	17.6%
[50]	cross-sectional	309 M/W 62.7 years	25.3 ± 2.9	6.7 ± 1.1	SMI	24.3%
[51]	cross-sectional	318 M/W 72.2 years	24.1 ± 4.4	7.3 ± 1.1	SARC-F	22.5%
[52]	cross-sectional	146 M/W 72.6 years	23.7 ± 3.6	7.1 ± 0.7	AWGS	14.4%
[53]	cross-sectional	391 M/W 72.4 years	23.8 ± 4.4	7.5 ± 1.4	AWGS	14.1%
[54]	cross-sectional	433 M/W 65.4 years	25.0 ± 4.7	7.6 ± 1.6	AWGS	7.4%
[55]	retrospective	312 M/W 64 years	24.7 ± 4.2	7.3 ± 1.4	AWGS	18%
[56]	cross-sectional	132 M/W 75.7 years	24.5 ± 3	7.8 ± 2.8	AWGS	28.8%
[57]	cross-sectional	746 M/W 69.9 years	24.7 ± 4.1	7.4 ± 1.3	AWGS	7%
[58]	cross-sectional	387 M/W 68.3 years	-	-	AWGS	27.4%
[59]	cross-sectional	166 M/W 63.2 years	26.3 ± 4.9	7.1 ± 1.1	AWGS	7.2%
[60]	cross-sectional	204 M/W 71.8 years	23.8 ± 4.0	7.2 ± 1.0	AWGS	8.8%
[61]	cross-sectional	183 M/W 64.7 years	25.3 ± 4.7	9.5 ± 2.1	AWGS	22%
[62]	cross-sectional	285 M/W 66.2 years	25.0 ± 4.6	7.1 ± 1.0	AWGS	8.8%
[63]	retrospective	716 M/W 65 years	24.7 ± 3.7	8.3 ± 1.9	AWGS	23.9%
[64]	cross-sectional	242 M/W 68.3 years	29.5 ± 4.5	7.8 ± 1.5	EWGSOP	21%
[65]	cross-sectional	250 M/W 71.7 years	25.3 ± 4.8	7.4 ± 1.0	SARC-F	19.5%
[66]	cross-sectional	267 M/W 73.7 years	24.0 ± 3.3	7.0 ± 1.0	AWGS	18.9%
[6]	cross-sectional	198 M/W 64.9 years	33.7 ± 7.7	8.1 ± 2.0	SARC-F	29.3%
[67]	cross-sectional	288 M/W 73.3 years	23.9 ± 3.5	7.6 ± 1.1	AWGS	15.2%

BMI: body mass index; HbA1c: glycated hemoglobin A1c; M: men; W: women; AWGS: Asian Working Group for Sarcopenia; SMI: skeletal muscle index; SARC-F: sluggishness, assistance in walking, rising from a chair, climb stairs, falls; -: data not available; EWGSOP: European Working Group on Sarcopenia in Older People.

**Table 2:** Prevalence of lifestyle habits in type 2 diabetes mellitus individuals with and without sarcopenia.

Reference	Smoking		Physical Activity		Habitual Alcohol Consumption	
	With Sarcopenia	Without Sarcopenia	With Sarcopenia	Without Sarcopenia	With Sarcopenia	Without Sarcopenia
[47]	5.8%	16.5%	58%	48.8%	-	-
[52]	42.8%	14.4%	57.1%	50.4%	-	-
[53]	3.6%	4.5%	12.7%	17.5%	-	-
[54]	12.5%	14.7%	6.3%	10.5%	-	-
[56]	26.3%	25.5%	52.6%	69.1%	15.7%	2.8%
[57]	-	-	42%	55%	-	-
[64]	0%	7%	-	-	46%	39%
[65]	18.4%	20.8%	-	-	13.5%	24.5%
[77]	26.3%	29.1%	37.8%	47.5%^*^	10.3%	15.6%

* *p* < 0.05. -: data not available; T2DM: type 2 diabetes mellitus.

**Table 3:** Glucose-lowering drug utilization in type 2 diabetes mellitus individuals with and without sarcopenia.

Reference	Metformin		TZDs		DPP-4 Inhibitors		Sulphonylureas		SGLT-2 Inhibitors		GLP-1 Analogues		Insulin	
	With Sarcopenia	Without Sarcopenia	With Sarcopenia	Without Sarcopenia	With Sarcopenia	Without Sarcopenia	With Sarcopenia	Without Sarcopenia	With Sarcopenia	Without Sarcopenia	With Sarcopenia	Without Sarcopenia	With Sarcopenia	Without Sarcopenia
[49]	19%	54% *	7%	10%	52%	60%	33%	24%	0%	3%	0%	5%	31%	24%
[52]	19%	38.4%	0%	5.6%	66.6%	48%	47.6%	35.2%	0%	12%	-	-	28.6%	14.4%
[56]	13.2%	42.5% *	-	-	-	-	-	-	-	-	-	-	68.4%	74.5%
[57]	25%	37.8%	11.5%	15%	59.6%	60.2%	17.3%	27.5%	5.8%	13.8%	5.8%	5.2%	59.6%	71.8%
[65]	-	-	-	-	-	-	-	-	-	-	12.2%	8.9%	79.5%	62.1% *
[66]	6%	24.4% *	6%	7.3%	52%	48.8%	20%	27.1%	0%	0.9%	1.5%	1.4%	24%	34.6%
[77]	48.6%	57.6% *	11.1%	12.8%	1%	0%	-	-	-	-	-	-	28.7%	24.8%

* *p* < 0.05. TZDs: thiazolidinediones; DPP-4: dipeptidyl peptidase 4; SGLT-2: sodium-glucose cotransporters; GLP-1: glucagon-like peptide-1; -: data not available.
